# Can development of asthma and bronchial hyperreactivity be reduced by subcutaneous immunotherapy in adult patients with allergic rhinitis?

**DOI:** 10.55730/1300-0144.5643

**Published:** 2023-06-12

**Authors:** Fatma Merve TEPETAM, Cihan ÖRÇEN, Zeynep Ferhan ÖZŞEKER, Dildar DUMAN, Sema SARAÇ

**Affiliations:** 1 Department of Immunology and Allergy, Hamidiye Faculty of Medicine, University of Health Sciences, Süreyyapaşa Chest Diseases and Thoracic Surgery Training and Research Hospital, İstanbul, Turkey; 2 Derince Training and Research Hospital, Kocaeli, Turkey; 3 Department of Immunology and Allergy, Cerrahpaşa Faculty of Medicine, İstanbul University, İstanbul, Turkey; 4 Department of Pulmonology, Hamidiye Faculty of Medicine, University of Health Sciences, Süreyyapaşa Chest Diseases and Thoracic Surgery Training and Research Hospital, İstanbul, Turkey

**Keywords:** Allergic rhinitis, asthma, bronchial hyperreactivity (BHR), methacholine challenge, PC20, subcutaneous immunotherapy (SCIT)

## Abstract

**Background/aim:**

Allergic rhinitis can be associated with bronchial hyperreactivity (BHR) and create an increased risk for allergic asthma development. We aimed to investigate the effects of subcutaneous immunotherapy (SCIT) on BHR and asthma development in adult patients with allergic rhinitis.

**Material and methods:**

The retrospective case-control study was carried out between November 2018 and May 2019 in Süreyyapaşa Chest Diseases and Thoracic Surgery Training and Research Hospital. In this study, data was recorded for patients with a mite and/or grasses/cereals pollen allergy who were tested for BHR before planned SCIT, and who had allergic rhinitis, with or without asthma. The SCIT group was selected as those who received SCIT for at least one year. The control group was selected from those who were scheduled to receive SCIT but were waived and still receiving medication. Symptom scores, prick test results, PC20 levels (methacholine challenge that is a provocative concentration causing a 20% fall in FEV1), and the presence of asthma were recorded and compared with data from at least one year after treatment.

**Results:**

A total of sixty-eight subjects (22 males, 46 females; mean age 40.54 ± 12.27 years; SCIT: 40, Control: 28) were enrolled. Although the changes in log PC20 levels were not statistically significant in both SCIT and control groups after an average of 30–35 months of treatment, it was found to be significant in favor of the SCIT group when two groups were compared in terms of the change in log PC20 (p = 0.026). The development and improvement of asthma were not significantly different between the SCIT and control group but tended to increase in the control group. The percentage of patients with progressed/BHR was significantly higher in the controls (70.6% vs. 38.1%, p = 0.046).

**Conclusion:**

In our real life study we have demonstrated the preventative effect of SCIT on BHR, but not on asthma development.

## 1. Introduction

Allergic rhinitis (AR) can be associated with bronchial hyperreactivity (BHR) and create an increased risk for asthma development [1]. Nine-year follow-up data from the European Community Respiratory Health Survey (ECRHS) showed that 9.7% of patients with AR had newly developed BHR, compared with 5.5% of subjects without AR [2]. It was demonstrated that small airway obstruction has been constituted a relevant predictive factor for severe BHR, in patients with AR [3]. Of the patients with AR, 34%–50% complained of occasional lower airway symptoms, such as wheezing, dyspnea, coughing, or chest tightness, while 14%–58% of the AR patients with asymptomatic BHR developed symptomatic asthma [4,5]. Asthma symptoms or BHR in patients with AR could be indicative of undiagnosed asthma.

Subcutaneous immunotherapy (SCIT) is used for allergic rhinitis (AR) and allergic asthma (AA) due to sensitization to aeroallergens such as pollens and house dust mites (HDM). Well-controlled clinical trials have demonstrated its efficacy in reducing symptoms and medication scores [6–8]. A large, prospective, randomized, controlled preventative allergy treatment (PAT) study conducted in children, confirmed a preventative effect of SCIT for asthma development [9]. There have been very few studies in adults regarding the preventative, or reductive effects of SCIT compared with traditional medical treatments. Only a few studies have investigated the preventative effects of immunotherapy in adults, and the subjects selected were monosensitized, without asthma or asthma-like symptoms. The first study was a double-blind, placebo-controlled trial, conducted in 2000 with 22 adults to evaluate BHR and the prevention of asthma. They found that SCIT increased the provocative dose of the methacholine challenge (where a provocative dose causes a 20% fall in FEV1 [PD20]) by 2.88 fold after one year, and none of the patients presented with asthma after 2 years of treatment [10]. Another study on 75 adult patients was an open observational study that showed that after five years of treatment, PD20 levels were significantly lower in the controls than in the sublingual immunotherapy (SLIT) group [11].

The potential preventative and reductive effects of SCIT in adults have never been studied in a real-life setting where the subjects are both mono or polysensitized, and with or without asthma. We aimed to investigate the effect of SCIT on BHR and asthma development, our secondary goal was to investigate new sensitization and any changes in peripheral eosinophil levels as an inflammation parameter.

## 2. Methods

### 2.1. Study design

The retrospective case-control study was carried out between November 2018 and May 2019 in Süreyyapaşa Chest Diseases and Thoracic Surgery Training and Research Hospital immunology and allergy department. The study was approved by the internal ethical committee of our hospital.

**Figure 1:**
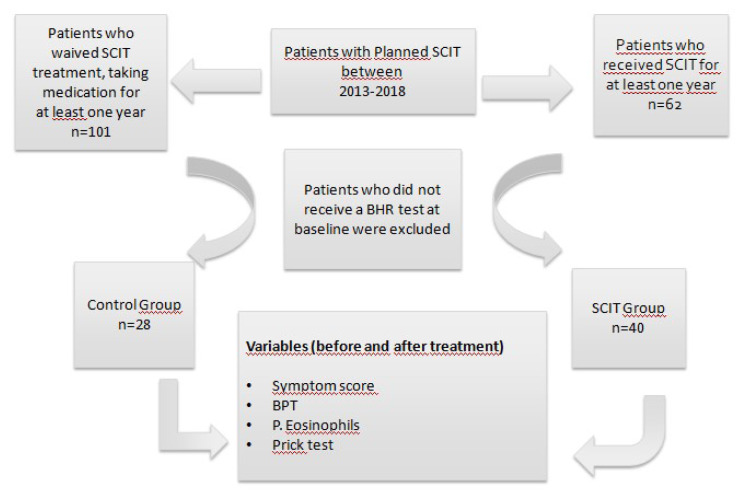
Flow chart of the study population. Abbreviations: SCIT, subcutaneous immunotherapy; BHR, bronchial hyperreactivity, BPT: bronchoprovocation test.

Data was recorded for those patients with a mite and/or grasses and cereals allergy who were evaluated for BHR with symptoms of allergic rhinitis, with or without asthma, prior to planned SCIT. Patients in the SCIT group were selected as those who had received SCIT for at least one year, in addition to other medication, between 2013–2018. The control group included those patients who were to receive SCIT, but for various reasons (lack of time, change of residency, fear of adverse reactions, or economic status) were not able to receive immunotherapy and also were on other medication. Exclusion criteria included, patients with other aeroallergen sensitivity, not being tested for BHR at baseline, other causes of BHR, chronic obstructive pulmonary disease (COPD), upper airway infections and bronchiectasis, and receiving SCIT previously. Symptom scores, prick test results, BHR, PC20 levels, and eosinophil levels, before treatment and after at least one year of treatment, were recorded (in our Immunology and Allergy department, besides evaluating clinical outcomes, we repeat the prick test and BHR tests after a one-year period for all patients receiving SCIT, and all those who have been scheduled to receive SCIT) (see Figure 1 for a flow chart of the study design).

### 2.2. Symptom score

For the clinical symptoms score, before and after treatment, patients attributed a daily score to their nasal symptoms, i.e. nasal obstruction, sneezing, nasal itching, and runny nose, and ocular symptoms, i.e. itching, redness, tearing (eye watering), 4-point scoring, from 0 up to 3 was applied for each of the nasal and ocular symptoms.; 0, no symptoms; 1, slight symptoms; 2, moderate symptoms; 3, severe symptoms, affecting their social life and work. The total score ranged from 0 to 21. Although the nasal and ocular symptom scores in the records were evaluated, the respiratory and drug scores could not be evaluated because they were not applied.

### 2.3. Measurement of bronchial hyperreactivity (BHR)

The 5-breath dosimeter method (Koko dosimeter, nSpire Health, Longmont, Colorado) was used according to the published guidelines from the American Thoracic Society [12]. Briefly, the methacholine bronchoprovocation test (BPT) sequence included five steps: diluent only at 0.0625, 0.25, 1.0, 4.0, and 16.0 mg/mL. If the cumulative concentration causing a 20% decrease in FEV1 (PC20 [provocative concentration] methacholine) was ≤16 mg/mL, the methacholine challenge test was evaluated to be positive for BHR.

### 2.4. Skin prick test

Aeroallergens (SoluprickSQ, ALK-Abello’ A/S, Hørsholm, Denmark) were used for the skin prick tests (SPT) and included positive histamine and negative (saline solution) controls, house dust mites (HDM), *Dermatophagoides farinae* (Der f) and *Dermatophagoides pteronyssinus* (Der p), grasses, cereals, tree mix, *Olea*, *Parietaria*, *Artemisia*, *Alternaria*, *Cladosporium herbarium*, *Aspergillus*, *Blatella germenica*, cat and dog dander. The puncture method with a 1 mm disposable lancet tip was used and a mean wheal diameter of 3 mm or greater than the control was considered positive. Both groups of patients were only sensitive to HDM and/or grasses and cereals prior to treatment. Patients with sensitivity to only HDM, or grasses and cereals, were regarded as monosensitized, while sensitivity to both of the two aeroallergens was regarded as polysensitization. If a patient became sensitive to any of the other aeroallergens after treatment they were categorized with new sensitization.

### 2.5 Subcutaneous immunotherapy

Cluster SCIT was performed with standardized allergen extracts from Der p and Der f (50–5000 therapeutic units (TU)/mL, aluminum hydroxide adsorbed Novo-Helisen Depot, (Allergopharma Joachim Ganzer KG) or 100–100,000 standardized quality units (SQ-U)/mL; Alutard SQ, (Hørsholm, Denmark). Application of preseasonal Allergovit (1000–10,000 TU/mL, Allergopharma), including allergoid grass and cereal extracts, was initiated three months prior to pollen season. While deciding which allergen to perform SCIT with in polysensitized patients, the major allergen responsible for the clinical symptoms, as determined by the history was taken into account. Both schedules were administered in hospital, including a 6–7-week induction phase with weekly injections, and a maintenance phase lasting at least three years, with monthly administrations. The patients were kept under observation for 30 min after each injection.

### 2.6. Medication history

Medication history was recorded from the data file if not documented in the hospital system pharmacy casting or social security institution. Recorded medications included nasal and oral antihistamines (AH), nasal and inhaled corticosteroids (IKS), short-acting beta agonists, montelukast (M), montelukast+antihistamine, inhaled corticosteroid+long-acting beta agonists (LABA).

### 2.7. Eosinophil levels

Peripheral blood eosinophil levels were determined using a Coulter LH 780 Hematology Analyzer (Beckman Coulter Inc., Brea, CA, USA).

### 2.8. Defining asthma and its severity

As stated in the Global Initiative for Asthma (GINA) 2021 report [13], asthma was diagnosed by demonstrating respiratory symptoms such as cough and wheezing, which change in severity and intensity over time, and variable airflow limitation demonstrated with bronchodilator reversibility test or BPT (PC20 level ≤ 8 mg/mL). All ongoing use of medications was recorded for each patient, and asthma severity was defined based on the treatment intensity. Patients receiving the treatment corresponding to the stages of 1–2 (i.e low dose IKS) were classified as mild, those receiving 3 or 4 stages of treatment (e.g, low or medium dose IKS-LABA) were defined as moderate asthma.

### 2.9. Asthma development

If a patient before treatment had no variable airflow limitation (FEV1/FVC < 80% or PC20 level ≤ 8 mg/mL), with or without any asthma symptoms such as wheezing, dyspnea, cough, or chest tightness triggered by exercise, allergens, or cold air, but had at least two of these symptoms and these spirometric findings after treatment, we evaluated it as development of asthma, according to guidelines [13–15].

### 2.10. Asthma improvement

If a patient had BHR (PC20 level ≤ 8 mg/mL), at baseline, but not after treatment (PC20 level >8 mg/mL or not provoked) we termed it as an improvement.

### 2. 11. Progression of/to BHR

**Figure 2:**
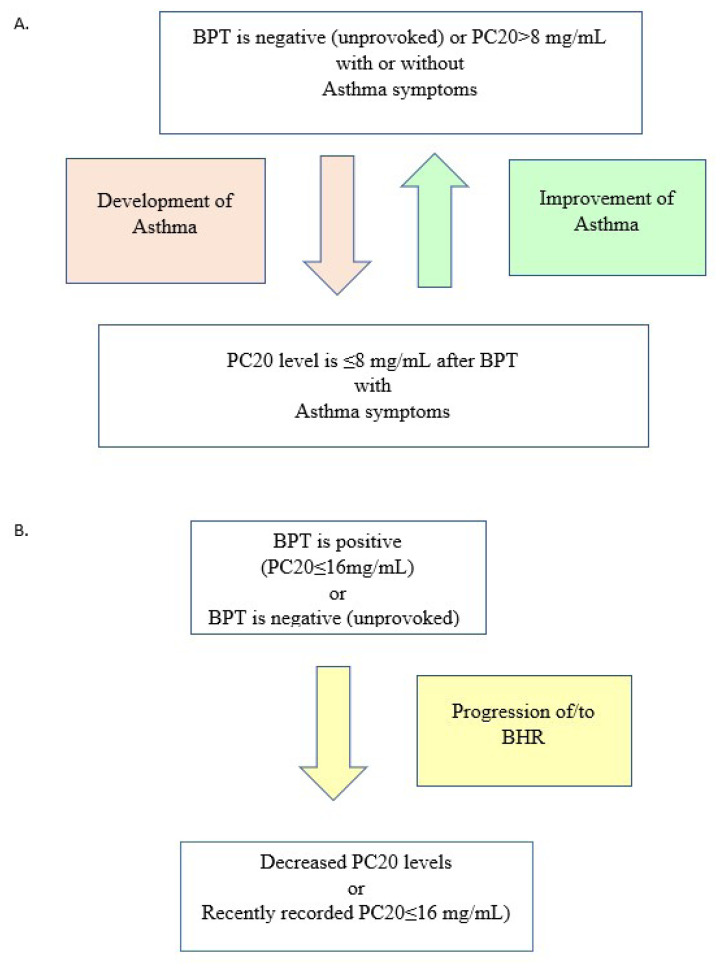
Description of asthma development improvement (A), progression of/to BHR (B). Abbreviations: BHR, bronchial hyperreactivity; PC20, methacholine challenge that is a provocative concentration causing a 20% fall in FEV1.

BPT is considered positive if PC20 level is less than or equal to 16 mg/mL. If these patients’ PC20 levels decreased, it was termed as the progression of BHR, if BPT was recently recorded as positive, (despite having normal bronchial responsiveness at baseline); was termed as progression to BHR (development of BHR) (Figure 2).

### 2.12. Statistical analyses

The mean ± SD values were given for normal distribution, and median values were given, as well as the 25%–75% percentile due to abnormal distribution. Gender equality, smoking, asthma, and mono/polysensitization in the different groups at baseline were tested using the chi-square test; for continuous parametric variables the Student’s t-test was used, and for nonparametric variables the Mann-Whitney U test was used. For posttreatment changes in symptom score and eosinophil percentage, the paired sample t-test was used because of normal distribution. The Wilcoxon signed rank test was used for changes in PC20 levels and eosinophil levels due to abnormal distribution. For assessing the onset of asthma and developing, the progressive disease we used the chi-square test. Multiple linear regression analysis was performed in order to identify factors (smoking status, age, gender, and polysensitization) that may be associated with the level of PC20. Since the distribution of PC20 was not normal, unmeasured values were given as 20 mg/mL (maximum PC20 level was 16 mg/mL) and calculated as the log PC20. Changes in bronchial responsiveness after treatment were calculated as the logarithm of the difference between log PC20 levels after treatment and baseline log PC20 levels, with a 95% confidence interval (CI). Since the Log PC20 is normally distributed, the paired t-test was used for posttreatment changes. Independent samples t-test was used for comparing the groups in terms of the change in log PC20 after treatment. A p-value < 0.05 was accepted as statistically significant.

## 3. Results

A total of sixty-eight subjects (22 males, 46 females; mean age 40.54 ± 12.27 years) with allergic rhinitis were enrolled in the study. The SCIT group comprised 40 patients and the control group included 28 patients. In the SCIT group, 45% of the patients had polysensitization, compared with 32% in the control group. Of the 40 patients in the SCIT group, six received Allergovit including an allergen extract of grasses and cereals, 31 received HDM, and three received immunotherapy with both allergens. Considering the level of clinical relevance, HDM was applied to 10 of 18 polysensitized patients, while SCIT-containing pollen (grasses-cereals) was applied to 5 of them. There were no statistically significant differences in terms of mean age, gender, smoking history, duration of treatment, frequency of asthma, asthma-like symptoms, and mono-polysensitization between the groups. However, the baseline symptom score was significantly higher, and PC20 levels were significantly lower, in the SCIT group (Table 1).

**Figure 3:**
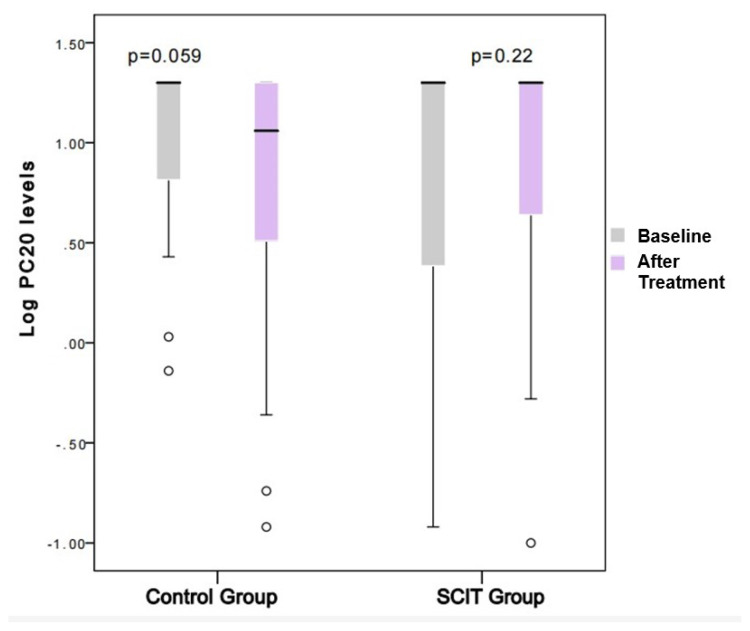
Changes in log PC20 levels after treatment in the SCIT and control groups. Since the Log PC20 is normally distributed the paired t-test was used for posttreatment changes. Abbreviations: SCIT, subcutaneous immunotherapy; PC20, methacholine challenge that is a provocative concentration causing a 20% fall in FEV1.

**Figure 4:**
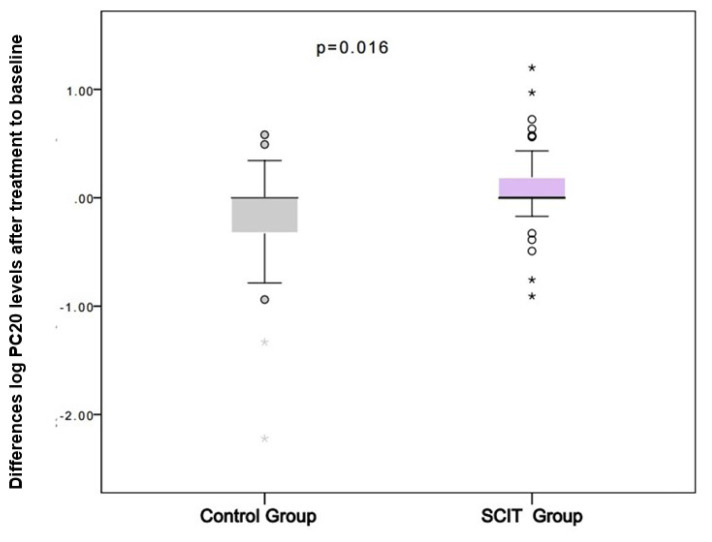
Comparison of differences in log PC20 levels from baseline to posttreatment between the SCIT and control group. Independent samples t-test was used. Abbreviations: SCIT, subcutaneous immunotherapy; PC20, methacholine challenge that is a provocative concentration causing a 20% fall in FEV1; log PC20, since the distribution of PC20 was not normal, unmeasured values were recorded as 20 mg/mL and calculated as the logarithm.

While symptom scores and eosinophils were significantly decreased in the SCIT group, PC20 levels, and eosinophil percentages were not significantly different between the two groups after treatment (Table 2). The logarithmic changes in PC20 levels were, however, increased in the SCIT group and decreased in the control group (Figure 3). Smoking status, age, gender, and polysensitization did not affect PC20 values in the linear logistic regression model. Although the changes in log PC20 levels did not reach statistical significance in both SCIT and control groups after an average of 30–35 months of treatment, it was significant in favor of the SCIT group when the groups were compared in terms of the change in log PC20 after treatment (Figure 4, p = 0.026).

### 3.1. Asthma severity

Considering the baseline treatment steps of asthmatic patients, twelve of 15 asthmatic patients in the SCIT group were evaluated as moderate (80%) and three of them were mild asthma (20%), while four of 8 asthmatic patients in the control group were evaluated as moderate (50%) and the remaining four patients were mild (50%).

In the follow-up, drug compliance was very poor in both groups. The prescribed drugs (IKS/IKS-LABA) in the SCIT and control groups during the last 1 year of treatment were similar when examined from the hospital registry system (median number of prescribed low dose IKS; as in number of boxes; SCIT group: 1, Control group: 1, p = 0.78; for low-medium IKS/LABA; SCIT group: 3, control group: 6, p = 0.33).

### 3.1. Asthma development

The onset of asthma was seen in one of 25 patients (4%) in the SCIT group, but four of 20 patients (20%) with no asthma at baseline in the control group developed asthma (p = 0.22). Both groups had five patients with asthma-like symptoms but no variable airflow limitation detected by bronchodilator reversibility test or BPT at baseline.

### 3.3. Asthma improvement

On the other hand, of the 15 asthmatic patients in the SCIT group, three (20%) improved (PC20 level exceeded 8 mg or not provoked), while three (37.5%) of the eight asthmatic controls had improved disease status (p = 0.22).

### 3.4. Progression of/to BHR

**Figure 5:**
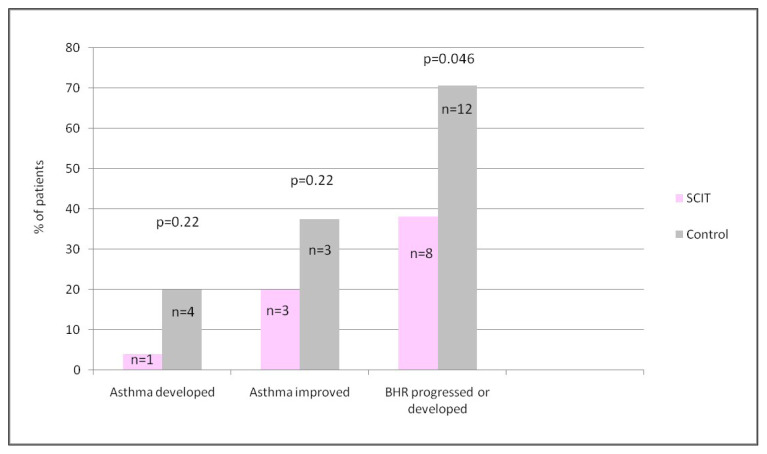
The preventative effect of subcutaneous immunotherapy on the progression or development of BHR, but not the development or improvement of asthma. Abbreviations: SCIT, subcutaneous immunotherapy; BHR, bronchial hyperreactivity, n, number of patients in the specified group.

When the methacholine provocation test positivity (PC20 ≤ 16 mg/mL) was evaluated, there were 17 patients in the SCIT group and 12 patients in the control group tested positive at baseline. The number of patients who turned positive, was four, in the SCIT group and five in the control group. Among patients with altered BHR, the percentage of patients with progressed PC20 level or a newly onset BHR (PC20 ≤ 16 mg/mL) was significantly higher in the control group (12/17; 70.6% vs. 8/21; 38.1%, p = 0.046) (Figure 5).

There were no significant differences in terms of medications used (except antihistamines) between the groups. Oral antihistamines were widely used in the SCIT group (p = 0.029).

### 3.5. New sensitization

In both groups, only two polysensitized patients had new sensitization. In the SCIT group; trees mix, *Parietaria*, and in the control group; *Olea europae*, *Alternia*.

## 4. Discussion

In our study, we showed that in adult AR patients, SCIT not only reduced the nasal and ocular symptom scores but also increased the log PC20 value, prevented the progression of/to BHR, and decreased eosinophil levels. There were, however, no significant differences in terms of new sensitization, or asthma development and improvement between the groups.

Consistent with other studies [10,11], our study showed the reductive effects of SCIT on BHR in terms of raising the PC20 value. However, in our study, the development or improvement of asthma was not significantly different between the SCIT and control group. Although the onset of asthma tended to increase in the control group, improvement of asthma also appeared to be greater in the control group; which may be due to the baseline of the patients in the control being better in terms of PC20 level. While not significant, it may be important to note that if a patient does not have asthma at baseline, SCIT may prevent the development of asthma, but is difficult to improve asthma. Disease progression can, however, be prevented with increased PC20 values and decreased eosinophil levels after SCIT. A randomized controlled study evaluating the effectiveness of SLIT and taking asthma severity into account showed that the frequency of intermittent asthma tended to decrease in both groups after three years of treatment, but the development of persistent asthma was significantly higher in the control arm, who were receiving only medication [16]. Despite asthma development and improvement being evaluated in as little as 2.5 years in our study, there were two retrospective cohort studies up to six years follow up including pediatric and adult patients, evaluating the efficacy of allergen immunotherapy (first with HDM, second with birch pollen) in the treatment of allergic rhinitis and/or asthma. In both studies, it was observed that asthma progression was reduced, which was evaluated by taking into account the asthma drug use status. Although we determined the severity of asthma according to the treatment received by the patients at the base of our study, we did not take this into account in the evaluation of the progression, since the drug compliance of the patients was very poor in the follow-ups. There was no difference in terms of the asthma drugs used between the groups. Besides, the probability of asthma development assessed by using logistic regression, was significantly lower in the first study and a significantly decreased risk of new-onset asthma medication use was found in the second study [17,18]. However, evaluating only the use of medication to define the onset of the progression of asthma may have led to false negatives. Provocation tests, which are the most valuable method in the diagnosis of asthma, were evaluated in a meta-analysis. The effect of immunotherapy on asthma was evaluated, and results in favor of immunotherapy were found in the histamine provocation test, although there was no clear evidence in favor of immunotherapy in the methacholine provocation test for the benefit of nonspecific BHR as a secondary result [19]. We inspected two studies included in this meta-analysis; the first was conducted by Bahceciler et al. [20] and investigated the effect of SLIT on asthma. They found no significant change in PC20 levels with the methacholine provocation test, in both the placebo and SLIT arms, after six months. Bousquet et al. [21] on the other hand, found that posttreatment PD20 was 1.98 and 1.75 times higher than baseline after 11 and 25 months, respectively, in the SLIT group, although they found no statistically significant difference between the SLIT and placebo group in posttreatment PD20. In our study, although log PC20 changes in the SCIT group, and log PC20 changes in the control group, were not significant after an average of 30–35 months of treatment, the difference between the groups after treatment was significant in favor of the SCIT group. In a PAT study, bronchial responsiveness to methacholine showed no statistically significant difference between the active and control groups at the 10-year follow-up, in concordance with the five-year follow-up [9,15]. It was explained by a natural spontaneous improvement of bronchial responsiveness over time. All of these studies may indicate that SCIT may reduce BHR in the short-term (1–5 years), but the effect may be similar to the control group in the long-term following a natural improvement.

Conversely to other studies, we showed a decrease in peripheral eosinophil levels in the SCIT group, but we did not investigate ECP or bronchial fluid eosinophil levels, as the other studies showed an antiinflammatory effect of SCIT [22–24].

A preventative effect of immunotherapy on the onset of new sensitizations has been reported in position papers and consensus documents based on the findings from some studies, but there is not enough solid evidence for this. Higher quality, long-term follow-up studies are needed to verify this [25,26]. A recently published meta-analysis does not support the claim that immunotherapy prevents new sensitization [27]. In our study, only two patients in both groups had new sensitization (mostly pollens), similar to that described in other studies [28,29]. However, almost half of the patients in our study were polysensitized and new sensitizations were seen in polysensitized patients. One study has shown that new sensitization can be prevented by SCIT in monosensitized patients [29].

We have some study limitations, our study was retrospective, and not a randomized case control study, there is selection bias with the SCIT group being more symptomatic, and more severe in terms of BHR. We did not use a medication score; we investigated the natural course of patients whether they were taking medication or not. Since drug compliance was similarly low in both groups, the change in asthma severity level after treatment was not evaluated. Moreover, follow-up period of 30–35 months (i.e. 2.5 years), may be a short time to assess asthma development or improvement. The PC20 cut-off point was taken as 8 mg/mL for the diagnosis of asthma, while 16 mg/mL was taken for the evaluation of BHR. If we could use more steps for assessing BHR with a high concentration of methacholine (e.g., 32 mg/mL or two min tidal breathing method), the change in PC20 after treatment could have been better evaluated.

A strength of our study was that the patients were not selected on whether they had asthma or not, or were mono or polysensitized; their follow-ups were left to their natural course. This is the real life study in adults with AR regarding the preventive or reductive effects of SCIT. We showed that in adult patients, SCIT reduced BHR while raising log PC20 values, prevented progression of/to BHR, and decreased eosinophil levels. A randomized, prospective, controlled, longer follow-up study with more patients in adults is warranted.

## Figures and Tables

**Table 1:** Baseline characteristics of SCIT and control patient groups.

	SCIT (n = 40)	Control (n = 28)	p-value

Age, years (mean)	39 ± 12	43 ± 13	0.21
Male, n (%)	29 (72)	17 (60)	0.43

Smoking history			
Continuous n (%)	5 (12.5)	5 (17.9)	0.14
Packet/year mean, SD	15 ± 10	12 ± 7	0.61

Asthma, n (%)	15 (37.5)	8 (28.5)	0.30

Asthma-like symptoms, n (%)	22 (55)	17 (60.7)	0.80

Prick test, n (%)			
Grasses-cereals	1 (2.5)	4 (14.3)	0.14
Der p + Der f	21 (52.5)	15 (53.6)	
Grasses-cereals + Der p + Der f	18 (45)	9 (32.1)	

FEV1, lt	3.10 ± 0.70	3.03 ± 0.59	0.4

Duration of treatment, months	34.6 ± 11.2	30.1 ± 8.8	0.08

Symptom score	15.2 ± 3.8	10.65 ± 5.57	<0.001

Eosinophil, %	4.2 ± 2.6	2.79 ± 1.23	0.41
Eosinophil, cell/mL median (IQR)	260 (120–340)	110 (100–310)	0.43

PC20, mg/mL median (IQR)	1.85 (0.7–3.2)	5.53 (3.3–9.5)	0.024

Abbreviations: SCIT, subcutaneous immunotherapy; PC20, methacholine challenge that is a provocative concentration causing a 20% fall in FEV1; Der p, *Dermatophagoides pteronyssinus*; Der f, *Dermatophagoides farina*; IQR, interquartile range.

P < 0.05: statistically significant

**Table 2:** Changes in symptom score, eosinophil, PC20, and log PC20 levels after treatment in both SCIT and control groups.

	SCIT n = 40			Control n = 28		
	Baseline	35 month+	p value	Baseline	30 month+	p value
Symptom Score*	15.3	6.8	<0.001	10.6	8.9	0.061
Eosinophils (cell/mL)^	260	200	0.023	110	100	0.59
Eosinophils (%)*	4.1	3.4	0.057	2.8	2.6	0.72
PC20 (mg/mL)^	1.85	3.76	0.078	5.53	5.71	0.85
Log PC20 (mg/mL)*	0.84	0.91	0.22	1.02	0.81	0.059

* Mean values were given for normal distribution. The paired t-test was used for posttreatment changes.

^ Median values were given for abnormal distribution. The Wilcoxon signed rank test was used for posttreatment changes.

+ Mean duration of treatment. P < 0.05 statistically significant

Abbreviations: SCIT, subcutaneous immunotherapy; PC20, methacholine challenge that is a provocative concentration causing a 20% fall in FEV1; log PC20, since the distribution of PC20 was not normal, unmeasured values were recorded as 20 mg/mL and calculated as the logarithm.
